# Moderating Effect of Psychosocial Safety Climate on the Association of Job Demands and Job Resources With Psychological Distress Among Japanese Employees: A Cross-sectional Study

**DOI:** 10.1016/j.shaw.2025.02.001

**Published:** 2025-02-03

**Authors:** Akiomi Inoue, Hisashi Eguchi, Yuko Kachi, Akizumi Tsutsumi

**Affiliations:** 1 Institutional Research Center, University of Occupational and Environmental Health, Japan, Kitakyushu, Japan; 2 Department of Mental Health, Institute of Industrial Ecological Sciences, University of Occupational and Environmental Health, Japan, Kitakyushu, Japan; 3 Department of Public Health, Kitasato University School of Medicine, Sagamihara, Japan

**Keywords:** Amplifying effect, Buffering effect, Job demands-resources model, Primary prevention, Psychosocial risks

## Abstract

**Background:**

We examined the moderating (buffering or amplifying) effect of psychosocial safety climate (PSC) on the association of job demands (psychological demands) and job resources (job control, supervisor support, coworker support, and extrinsic reward) with psychological distress among Japanese employees.

**Methods:**

A self-report web-based questionnaire was administered to 2,200 employees (1,100 men and 1,100 women) registered with a Japanese private online survey company. The questionnaire included scales on job demands and job resources (the Job Content Questionnaire and the short-form Effort–Reward Imbalance Questionnaire), PSC (the 12-item PSC scale), and psychological distress (the K6 scale) and items on participants' demographic and occupational characteristics (age, gender, education, occupation, work form, and working hours per week). Hierarchical multiple regression analyses were performed using psychological distress as a dependent variable. Interaction terms of job demands and job resources with PSC were included.

**Results:**

There was a significant interaction effect of psychological demands with PSC on psychological distress (*β* = −0.053, *p* = 0.008), adjusted for demographic and occupational characteristics. *Post hoc* simple slope analysis showed that the simple slope of psychological demands was lesser at higher levels of PSC (1 standard deviation above the mean) (*β* = 0.101, *p* < 0.001) than at lower levels (1 standard deviation below the mean) (*β* = 0.199, *p* < 0.001). No significant interactions were observed between job resources and PSC.

**Conclusion:**

Our findings suggest that PSC buffers the positive association of psychological demands with psychological distress.

## 1 Introduction

Psychosocial safety climate (PSC) theory has arisen as a recent framework for comprehending job stress, assisting in elucidating the genesis of stressful job attributes [1]. Abundant epidemiological studies have accumulated findings on the consequences of PSC for employee health over the past decade [2]. PSC, an organizational factor, is predominantly shaped by management and described as employees' shared perceptions of the organization's “policies, practices, and procedures for the protection of employee psychological health and safety” [1]. In particular, it focuses on whether (i) management shows support, engagement, and dedication to stress prevention efforts (management commitment), (ii) management places greater emphasis on psychological health and safety of employees rather than focusing solely on productivity objectives (management priority), (iii) the organization values input from employees on factors impacting psychological health (organizational commitment), and (iv) there is engagement and active involvement on health and safety matters from all levels of the organization (organizational participation) [3].

Consequences of the lack of PSC for psychological health problems among employees are often interpreted within a theoretical context of the extended job demands-resources (JD-R) model [1]. This model assumes that insufficient PSC leads to increased job demands (psychological demands, emotional demands, job insecurity, role ambiguity, role conflict, harassment, etc.) and decreased job resources (job control, worksite support, extrinsic reward, performance feedback, procedural fairness, opportunities for development, etc.), which, in turn, leads to a deterioration in psychological health problems. The relationship between PSC and job demands, as well as PSC and job resources, is a key mechanism within this model. As proposed by Dollard and Bakker [1], a lack of PSC reflects an organizational climate where psychological health is not prioritized, leading to poorly managed job demands and insufficient support systems. For example, in organizations with low PSC, employees may face excessive workloads or unclear role expectations due to inadequate workload management policies or insufficient communication from the management [1]. Additionally, emotional demands, such as the pressure to suppress emotions or handle interpersonal conflicts without adequate support, may increase in environments where psychological health is not actively safeguarded [4]. Simultaneously, insufficient PSC reduces job resources by limiting opportunities for employees to exercise control over their work, develop skills, or receive constructive feedback [5]. Mechanisms contributing to this include a lack of participatory decision-making, unclear performance expectations, and insufficient investment in employee development programs [6]. Moreover, the absence of organizational practices to recognize and reward employees' efforts exacerbates the decline in job resources, leaving employees ill-equipped to manage job demands [7]. By increasing job demands and limiting job resources, low PSC creates a work environment that significantly impacts employees' psychological health. Abundant epidemiological studies have fully or partially substantiated the assumptions of the extended JD-R model, particularly in Australia [1,[8], [9], [10], [11], [12], [13], [14]] and Malaysia [5,10,[15], [16], [17], [18], [19]], and a small number of similar findings have been observed in other countries [[20], [21], [22]]. Also, in Japan, our previous cross-sectional study reported the association of PSC with psychological distress and the mediating roles of job demands and job resources on it [23].

Thus, PSC has a primary role in predicting job demands and job resources and their impact on psychological health problems among employees. On the other hand, another role of PSC is supposed to moderate (buffer or amplify) the consequences of job demands and job resources for psychological health problems. Dollard and Bakker [1] have argued that, according to the conservation of resources theory [24], employees are more likely to be provided with supportive practices that lead to the conservation and accumulation of personal resources, such as the opportunity to debrief after emotionally challenging experiences, in high-PSC contexts. This could enhance their coping skills and buffer the consequences of job demands for psychological health problems. Furthermore, in such contexts, the impact of low personal resources is offset through a compensation process (additional support or resources are provided to the individual in the form of recognition, etc.), which may amplify the consequences of job resources for reducing psychological health problems [1]. Prior studies have consistently found that PSC buffers the consequences of job demands for psychological health problems [1,8,[25], [26], [27]]. Conversely, consistent findings on the amplifying effect of PSC on the consequences of job resources for reducing psychological health problems are lacking owing to limited studies [1,19]. Therefore, further studies are required.

In addition, the concept of PSC remains relatively unfamiliar in Japan, and the only Japanese findings on PSC and employee health are from our aforementioned study [23]. With its prevalence in Japanese companies dedicated to Health and Productivity Management, the concept of PSC is highly relevant. Hence, expanding on the findings of PSC and employee health could amplify the diffusion of its concept and advancement of occupational health initiatives across Japanese companies. However, the applicability of prior research findings to Japanese companies, which have distinct cultural backgrounds compared to Western and other Asian countries, remains unclear. Therefore, replication of prior findings is crucial to enhancing comprehension of PSC in Japanese companies.

This study aimed to examine the moderating (buffering or amplifying) effect of PSC on the association of job demands and job resources with psychological distress among Japanese employees, using the same database used in the aforementioned cross-sectional study [23]. In this study, we utilized the demand–control–support (DCS) [28] and effort–reward imbalance (ERI) models [29], which form the foundation of the JD-R model [30]. Hence, psychological demands were measured as job demands, while job control, supervisor support, coworker support, and extrinsic reward were measured as job resources. We hypothesized that PSC would buffer the positive association of job demands with psychological distress and amplify the negative association of job resources with psychological distress [1,19].

## 2 Materials and methods

### 2.1 Participants

As noted earlier, this study used the same database as our previously reported cross-sectional study [23]. In October 2020, a total of 42,784 men and 44,276 women were randomly selected from approximately 1.19 million people (570 thousand men and 620 thousand women) registered with a Japanese private online survey company. The randomly selected registrants were sent an advertisement offering online shopping points valued at a few hundred yen (equivalent to a few US dollars) as an incentive. Responses were handled on a first-come-first-served basis, and due to limitations in the project budget, recruitment ceased once the number of respondents reached 2,200. Respondents indicated whether they “currently worked” and “were employed by a company, organization, government office, or self-employed person or private household earning a salary or wage (including executives).” Because the online survey used a stratified sample, an equal number of participants were included in each age category (from 20s to 60s), maintaining a balanced male-to-female ratio of 1:1. All the variables were measured via a self-report web-based questionnaire, except age and gender, which were obtained from the online survey company's registration information. As the web-based questionnaire mandated participants to respond to every question, there were no instances of missing data. Participants' detailed characteristics and Pearson's correlation coefficients among the scale scores can be found in Table 1, Table 2, respectively. Participants were informed of the study objectives and protocols, and their informed consent was acquired electronically before the survey commenced. This study was reviewed and approved by the Kitasato University Medical Ethics Organization (No. B20-180).

**Table 1:** Participants' detailed characteristics

Demographic characteristics	Mean (SD)	*n* (%)
Age	44.6 (13.3)	
Gender		
Men		1,100 (50.0)
Women		1,100 (50.0)
Education		
Graduate school		126 (5.7)
College		1,054 (47.9)
Junior college		246 (11.2)
Vocational school		245 (11.1)
High school or junior high school		529 (24.0)
Occupation		
Managerial employee		249 (11.3)
Non-manual employee		1,508 (68.5)
Manual employee		294 (13.4)
Other		149 (6.8)
Work form		
Day shift		1,947 (88.5)
Shift work with night duty		184 (8.4)
Shift work without night duty		44 (2.0)
Night shift		25 (1.1)
Working hours per week		
30 hours or less		517 (23.5)
31–40 hours		725 (33.0)
41–50 hours		666 (30.3)
51–60 hours		184 (8.4)
61 hours or more		108 (4.9)

Scale scores	Mean (SD)	Cronbach *α*

Psychological demands (JCQ) (range: 12–48)	31.4 (5.89)	0.65
Job control (JCQ) (range: 24–96)	62.7 (11.2)	0.75
Supervisor support (JCQ) (range: 4–16)	10.5 (2.76)	0.91
Coworker support (JCQ) (range: 4–16)	10.9 (2.40)	0.87
Extrinsic reward (Short ERIQ) (range: 7–28)	17.4 (3.14)	0.68
Psychosocial safety climate (PSC-12) (range: 12–60)	34.8 (11.4)	0.97
Psychological distress (K6 scale) (range: 0–24)	6.77 (6.05)	0.94

Abbreviations: ERIQ, Effort–Reward Imbalance Questionnaire; JCQ, Job Content Questionnaire; PSC-12, 12-item psychosocial safety climate scale; SD, standard deviation.

**Table 2:** Pearson's correlation coefficients for age and scale scores

	1	2	3	4	5	6	7
1. Age							
2. Psychological demands (JCQ)	−0.176∗∗						
3. Job control (JCQ)	−0.007	0.083∗∗					
4. Supervisor support (JCQ)	−0.090∗∗	−0.201∗∗	0.330∗∗				
5. Coworker support (JCQ)	−0.115∗∗	−0.052∗	0.315∗∗	0.634∗∗			
6. Extrinsic reward (Short ERIQ)	−0.085∗∗	−0.179∗∗	0.276∗∗	0.517∗∗	0.443∗∗		
7. Psychosocial safety climate (PSC-12)	0.006	−0.167∗∗	0.324∗∗	0.606∗∗	0.494∗∗	0.488∗∗	
8. Psychological distress (K6 scale)	−0.242∗∗	0.278∗∗	−0.089∗∗	−0.229∗∗	−0.174∗∗	−0.339∗∗	−0.255∗∗

∗*p* < 0.05, ∗∗*p* < 0.01.

Abbreviations: ERIQ, Effort–Reward Imbalance Questionnaire; JCQ, Job Content Questionnaire; PSC-12, 12-item psychosocial safety climate scale.

### 2.2 Measures

#### 2.2.1 Exposures (job demands and job resources)

As mentioned previously, on the basis of the DCS and ERI models [28,29], psychological demands were measured as job demands, while job control, supervisor support, coworker support, and extrinsic reward were measured as job resources.

For job demands, psychological demands were measured via the Job Content Questionnaire (JCQ) Japanese version [31]. The JCQ includes a 5-item scale on psychological demands on a 4-point Likert scale (1 = *strongly disagree* to 4 = *strongly agree*). The total score (ranging from 12 to 48) was calculated in accordance with the JCQ User's Guide [32].

For job resources, job control, supervisor support, and coworker support were also measured via the JCQ [31]. Extrinsic reward was measured via the short form Effort–Reward Imbalance Questionnaire (Short ERIQ) Japanese version [33]. The JCQ includes a 9-item scale on job control, a 4-item scale on supervisor support, and a 4-item scale on coworker support on a 4-point Likert scale (1 = *strongly disagree* to 4 = *strongly agree*). Similar to psychological demands, the total scores (ranging from 24 to 96 for job control and from 4 to 16 for supervisor and coworker support) were calculated in accordance with the JCQ User's Guide [32]. The Short ERIQ includes a 7-item scale on extrinsic reward on a 4-point Likert scale (1 = *strongly disagree* to 4 = *strongly agree*). After reversing the scores corresponding to the items indicating unfavorable circumstances, the scores for each item were summed to calculate the total score (ranging from 7 to 28).

#### 2.2.2 Moderator: psychosocial safety climate

PSC was measured via the 12-item PSC scale Japanese version [34]. It assesses managerial attitudes and employee involvement in matters concerning psychological health and safety. Responses were rated on a 5-point Likert scale (1 = *strongly disagree* to 5 = *strongly agree*). The scores for each item were summed to calculate the total score (ranging from 12 to 60). The 12-item PSC scale Japanese version has been demonstrated to be a reliable and valid instrument for measuring PSC in Japanese workplaces, as evidenced by its high internal consistency (Cronbach α = 0.97), acceptable test–retest reliability [Cohen's weighted kappa = 0.53 and intraclass correlation (ICC) = 0.69], and structural validity supported by confirmatory factor analysis (e.g., goodness of fit index = 0.97, comparative fit index = 0.99, and root mean square error of approximation = 0.06).

#### 2.2.3 Outcome: psychological distress

Psychological distress was measured via the K6 scale Japanese version [35]. It assesses the levels of psychological distress, including feelings of nervousness (Q1), hopelessness (Q2), restlessness or fidgetiness (Q3), depression (Q4), effortfulness (Q5), and worthless (Q6) over the past 30 days. Responses were rated on a 5-point Likert scale (ranging 0 = *none of the time* to 4 = *all of the time*). The score for each item were summed to calculate the total score (ranging from 0 to 24).

#### 2.2.4 Covariates

Participants' demographic and occupational characteristics were measured as covariates. Demographic characteristics included age, gender, and education. Age was treated as a continuous variable. Education was categorized into 5 groups: graduate school, college, junior college, vocational school, and high school or junior high school. Occupational characteristics included occupation, work form, and working hours per week. Occupation was categorized into 4 groups: managerial, non-manual, manual employee, and others. Work form was also categorized into 4 groups: day shift, shift work with night duty, shift work without night duty, and night shift. Working hours per week were categorized into 5 groups: 30 hours or less, 31–40 hours, 41–50 hours, 51–60 hours, and 61 hours or more.

### 2.3 Statistical analysis

Hierarchical multiple regression analyses were performed using psychological distress (the total K6 score) as a dependent variable. Independent variables were entered in the following sequence: covariates (demographic and occupational characteristics) (Step 1), main effects of job demands (psychological demands), job resources (job control, supervisor support, coworker support, and extrinsic reward), and PSC (Step 2), and interaction terms of job demands and job resources with PSC (Step 3). If a significant interaction effect was observed in Step 3, a *post hoc* simple slope analysis was performed at 1 standard deviation (SD) above and below the mean PSC score. Throughout a sequence of analyses, *R*-squared (*R*^*2*^), adjusted *R*^*2*^, and *ΔR*^*2*^ (indicating an increase from the previous step) were calculated at each step to evaluate the model fit. Furthermore, a residual analysis was performed to evaluate the level of autocorrelation in the residuals via the Durbin–Watson statistic (ranging from 0 to 4.0 and a value of 2.0 indicated no autocorrelation). Prior to the analyses, job demands, job resources, and PSC scores were mean-centered to avoid multicollinearity when interaction terms were entered in Step 3. In addition to the total K6 score, separate analyses were conducted using the individual K6 item scores as dependent variables. This allowed for a more detailed investigation into how specific aspects of psychological distress (as captured by the K6) are associated with job demands, job resources, and PSC. The regression analyses followed the same procedure as for the total K6 score. The significance threshold was set at 0.05 (2-tailed). Statistical analyses were performed utilizing IBM SPSS Statistics for Windows (version 27.0; Armonk, NY: IBM Corp.).

## 3 Results

**Fig. 1:**
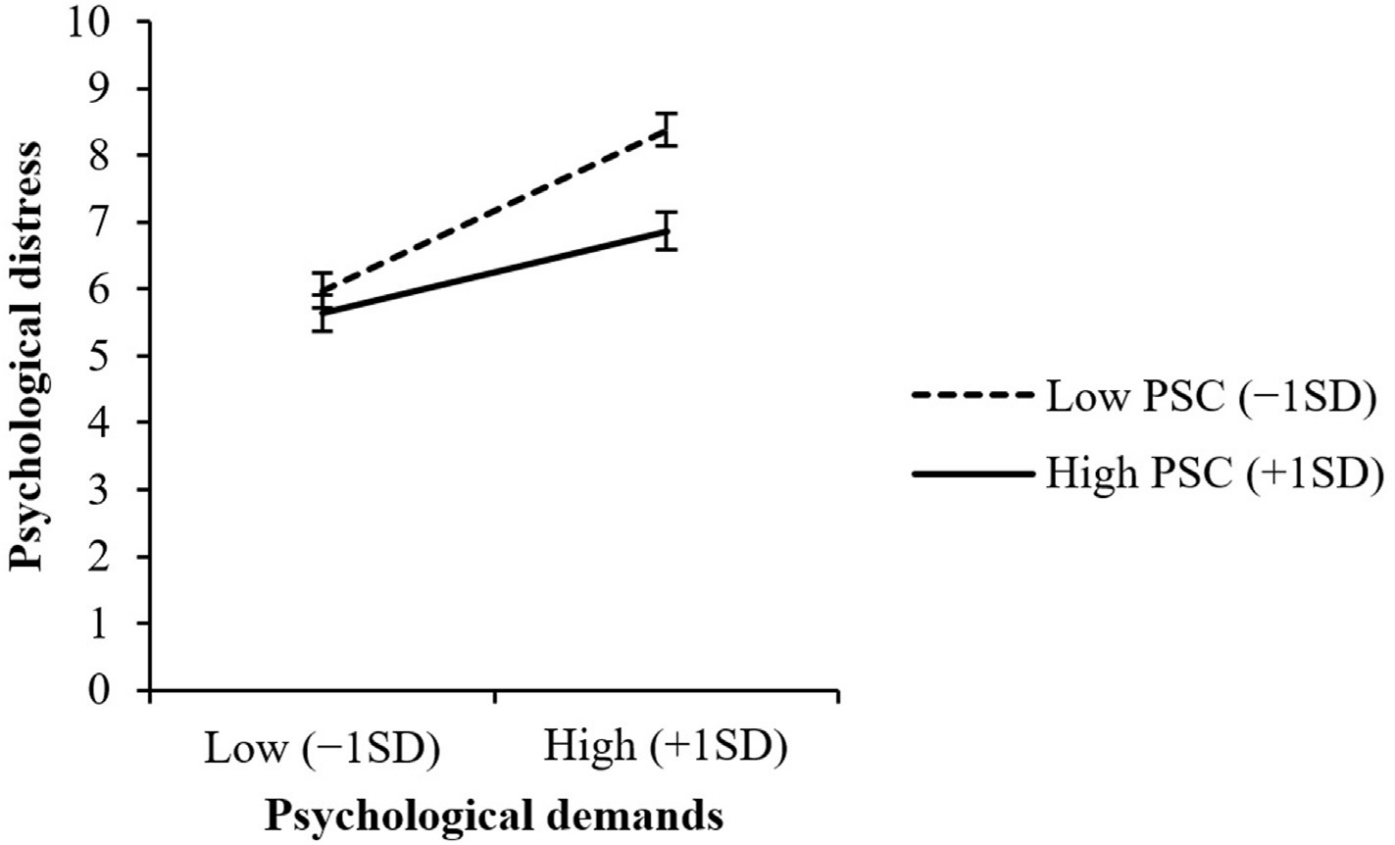
Interaction between psychological demands and psychosocial safety climate (PSC) on psychological distress: *post hoc* simple slope analysis. SD, standard deviation.

Table 3 presents the results of the hierarchical multiple regression analyses. There was a significant positive main effect of psychological demands (*β* = 0.159, *p* < 0.001) and significant negative main effects of extrinsic reward and PSC on psychological distress (*β* = −0.283, *p* < 0.001 and *β* = −0.077, *p* = 0.002, respectively), adjusted for demographic and occupational characteristics (Step 2). However, no significant main effects of job control, supervisor support, or coworker support were observed. When the interaction terms of job demands and job resources with PSC were added in the model (Step 3), main effects of psychological demands, extrinsic reward, and PSC remained significant (*β* = 0.150, *p* < 0.001; *β* = −0.280, *p* < 0.001; and *β* = −0.076, *p* = 0.003, respectively). Furthermore, a significant negative interaction effect of psychological demands with PSC was observed (*β* = −0.053, *p* = 0.008). However, job resources had no significant interactions with PSC. In addition, these interaction terms did not significantly contribute toward explaining psychological distress (*ΔR*^*2*^ = 0.003, *p* = 0.104). A *post hoc* simple slope analysis showed that the simple slope of psychological demands was lesser at higher levels of PSC (1 SD above the mean) (*β* = 0.101, *p* < 0.001) than at lower levels (1 SD below the mean) (*β* = 0.199, *p* < 0.001) (see Fig. 1). Residual analysis confirmed that the residuals were hardly autocorrelated as the Durbin–Watson statistics were extremely close to the optimal value of 2.0 (2.044, 2.019, and 2.021 for Steps 1–3, respectively).

**Table 3:** Associations of demographic and occupational characteristics, job demands, job resources, and psychosocial safety climate (PSC) with psychological distress: hierarchical multiple regression analyses

Standardized coefficient (*β*)	Step 1		Step 2		Step 3	
	Estimate	*p*	Estimate	*p*	Estimate	*p*
Age	−0.223	<0.001	−0.242	<0.001	−0.241	<0.001
Gender (men vs. women)	0.025	0.305	0.024	0.276	0.027	0.225
Education (vs. high school or junior high school)						
Graduate school	−0.022	0.350	0.000	0.983	−0.001	0.956
College	−0.021	0.431	0.021	0.387	0.019	0.447
Junior college	0.010	0.677	0.007	0.765	0.004	0.846
Vocational school	−0.026	0.274	−0.032	0.140	−0.034	0.119
Occupation (vs. managerial employee)						
Non-manual employee	0.026	0.443	−0.047	0.131	−0.047	0.131
Manual employee	0.030	0.322	−0.030	0.279	−0.033	0.247
Other	−0.003	0.904	−0.043	0.074	−0.045	0.064
Work form (vs. day shift)						
Shift work with night duty	0.028	0.188	0.023	0.246	0.023	0.245
Shift work without night duty	0.018	0.378	−0.007	0.705	−0.006	0.754
Night shift	0.014	0.494	0.000	0.983	0.000	0.983
Working hours per week (vs. 30 hours or less)						
31–40 hours	0.007	0.793	−0.012	0.624	−0.011	0.655
41–50 hours	0.048	0.092	−0.002	0.948	−0.001	0.982
51–60 hours	0.084	<0.001	0.042	0.062	0.042	0.063
61 hours or more	0.057	0.013	0.003	0.900	0.002	0.925
Psychological demands			0.159	<0.001	0.150	<0.001
Job control			−0.004	0.845	−0.007	0.766
Supervisor support			−0.017	0.554	−0.018	0.536
Coworker support			−0.021	0.412	−0.013	0.648
Extrinsic reward			−0.283	<0.001	−0.280	<0.001
Psychosocial safety climate (PSC)			−0.077	0.002	−0.076	0.003
Psychological demands × PSC					−0.053	0.008
Job control × PSC					−0.012	0.572
Supervisor support × PSC					−0.020	0.535
Coworker support × PSC					0.034	0.253
Extrinsic reward × PSC					0.003	0.888

Model fit indices	Estimate	*p*	Estimate	*p*	Estimate	*p*

*R*^*2*^	0.071	—	0.232	—	0.236	—
Adjusted *R*^*2*^	0.064	—	0.225	—	0.226	—
ΔR^*2*^	0.071	<0.001	0.161	<0.001	0.003	0.104
Residual analysis (Durbin–Watson statistic)	2.044		2.019		2.021

Separate analyses using the individual K6 item scores as dependent variables revealed consistent significant positive main effects of psychological demands (*β* = 0.069 to 0.187, *p* < 0.001 to 0.002) and significant negative main effects of extrinsic reward (*β* = −0.268 to −0.202, *p* < 0.001) across all six K6 items. Additionally, the negative interaction effect of psychological demands with PSC was significant for five items (Q2–Q6) (*β* = −0.055 to −0.043, *p* = 0.008 to 0.041) and marginally significant for nervousness (Q1) (*β* = −0.039, *p* = 0.065). These findings indicate that the results obtained using the total K6 score as the outcome variable were largely replicated when analyzing individual items. Detailed results of these item-specific analyses are provided in the Supplementary data.

## 4 Discussion

This study demonstrated a significant positive main effect of psychological demands and significant negative main effects of extrinsic reward and PSC on psychological distress. Furthermore, a significant negative interaction effect of psychological demands with PSC on psychological distress (i.e., buffering effect of PSC on the association of psychological demands with psychological distress) was observed. Job control, supervisor support, and coworker support had neither a significant main effect on psychological distress nor significant interaction with PSC.

For job demands, a significant positive main effect of psychological demands on psychological distress was observed. This is consistent with the “health impairment process,” in the JD-R model, which assumes that high job demands lead to strain and health impairment [36], as well as meta-analytic findings on the association of psychological demands with psychological health problems (common mental disorders and burnout) [37,38]. This study supported the theoretical model and replicated prior findings. Furthermore, there was a significant buffering effect of PSC on the association of psychological demands with psychological distress. This is also consistent with prior findings from Australia and Malaysia [1,8,[25], [26], [27]]. Our finding suggests that when workplaces have established systems and procedures to safeguard employees' psychological health and safety, the consequences of psychological demands for psychological health problems are mitigated. As a mechanism for buffering effect of PSC on job demands, Dollard and Bakker [1] have argued that in high-PSC contexts, employees' coping skills toward high job demands are enhanced through the provision of support to employees that leads to the conservation and accumulation of personal resources. Personal resources should also be included in future analytical models to substantiate their arguments and elucidate the mechanism in further detail.

For job resources, a significant negative main effect of extrinsic reward was observed. However, no significant main effects of job control, supervisor support, or coworker support were observed, which only partially supported the JD-R model that job resources improve psychological health problems [36]. As this study was conducted amid the coronavirus disease 2019 pandemic, a significant number of employees experienced economic hardship due to the pandemic. Furthermore, the widespread use of remote work made it difficult for companies to conduct fair and equitable personnel evaluations. Therefore, employees may have been more conscious of the fair reward allocation. It is also plausible that the extensive use of remote work reduced opportunities for in-person interactions with supervisors and coworkers or that the line between work and home life became blurred, even when job control increased, which may have resulted in an unclear association of job control, supervisor support, and coworker support with psychological distress. Furthermore, no significant interactions of job resources with PSC were observed. This is consistent with the findings of Dollard and Bakker [1]. However, the findings of Yulita *et al* are different [19]. Besides the fact that this study was conducted amid the coronavirus disease 2019 pandemic, discrepancy in these findings could be explained by differences in the indicators included in job resources. Dollard and Bakker [1] used mainly cognitive resources, such as job control, as indicators of job resources, while Yulita *et al* [19] included cognitive and emotional resources. Job control and extrinsic reward do not necessarily focus on the emotional domain of job resources [39,40]. Furthermore, items in the JCQ on supervisor and coworker support are not necessarily specific to measure the emotional domain [39]. Hence, a significant amplifying effect of PSC on the negative association of job resources with psychological distress may not have been observed in this study. Prior studies have suggested that PSC is possibly more aligned to an emotional resource rather than a cognitive one [1]. Therefore, future studies should focus on emotional resources and examine the moderating effect of PSC for each content domain of job resources.

This study has some limitations. First, this study was conducted exclusively among participants registered with a specific private online survey company. Therefore, our findings should be generalized with caution. Second, since PSC focuses on the “shared perceptions” of employees toward their organization, a multilevel analysis was theoretically required as it encompassed analysis at both individual and group levels [1]. However, owing to the non-nested structure of our sample within companies or workplaces, such an analysis could not be performed. Third, our findings may have been confounded by various factors that were not measured. For instance, individuals with higher levels of neurotic traits were more likely to assess their psychosocial work environment poorly [41] and may have been more psychologically distressed [42]. Hence, lack of measurements of personality traits may have caused an overestimation of the true association. Fourth, since the primary variables utilized in the analyses were measured via a self-report questionnaire, our findings may be susceptible to common method bias [43]. Fifth, this study captured only 5 factors as job demands and job resources according to the DCS and ERI models [28,29]. Therefore, future studies should measure various job demands (job insecurity, role ambiguity, role conflict, harassment, etc.) and job resources (performance feedback, procedural fairness, opportunities for development, etc.) to examine the moderating effect of PSC in further detail. Sixth, we did not obtain data on participants' employment status (e.g., full-time vs. part-time). In Japan, the standard working hours per week for full-time employees are typically 40 hours. While working hours per week were obtained, there was no clear and consistent classification of employment status, as some full-time employees may work fewer than 40 hours per week due to accommodations, such as health-related adjustments, and some part-time employees may work more than 40 hours per week due to overtime. This limitation prevents us from performing a subgroup analysis based on employment status, which may influence the generalizability of the findings. Lastly, owing to the cross-sectional design, causal inferences were limited. Our findings could indicate that individuals experiencing psychological distress tended to perceive their work as more demanding and their PSC as lower.

Considering these limitations, future research should focus on the following areas. First, longitudinal studies are needed to establish causal relationships between PSC, job demands, job resources, and psychological distress, particularly examining how PSC interacts with job demands and job resources over time to influence psychological distress. Second, intervention trials aimed at enhancing PSC could provide valuable insights into whether this enhancement buffers the consequences of psychological demands for psychological distress. Practical strategies for enhancing PSC may include management commitment, employee involvement in decision-making, and policies to reduce excessive working hours. Third, future research should consider subgroup analyses based on employment status as employment status may influence how PSC interacts with job demands and job resources, offering a more nuanced understanding of the factors contributing to psychological distress in different work settings. Lastly, research should consider industry-specific and cultural differences in the perception of PSC, as these factors could offer deeper insights into how PSC functions across various workplace contexts.

In conclusion, this study suggests that PSC buffers the positive association of psychological demands with psychological distress. However, the explanation rate for the interaction of job demands (and job resources) with PSC for psychological distress was not statistically significant. In addition, the simple slope of psychological demands was significant, even when PSC was high. Hence, to reduce the negative consequences of excessive psychological demands for mental health, reduction of psychological demands as well as enhancement of PSC is essential. In particular, addressing the issue of long working hours through organization-wide initiatives, such as the establishment of clear guidelines and policies to reduce working hours, along with the active involvement of management in monitoring and reducing workload, can substantially reduce psychological demands. Furthermore, to enhance PSC, it is essential that management demonstrates commitment to stress prevention, prioritizes psychological health and safety, and encourages participation from all levels of the organization [3]. Practical steps include ensuring regular check-ins between managers and employees, promoting work–life balance, and integrating employee feedback through surveys or structured feedback sessions. Fostering a culture of open communication, where employees feel supported in expressing concerns about stress and workload, is also crucial. Future research should explore if our results can be replicated through a prospective approach and conduct intervention trials to assess whether enhancing PSC buffers the consequences of psychological demands for psychological distress.

## CRediT authorship contribution statement

**Akiomi Inoue:** Writing – original draft, Visualization, Project administration, Methodology, Investigation, Funding acquisition, Formal analysis, Data curation, Conceptualization. **Hisashi Eguchi:** Writing – review & editing, Funding acquisition. **Yuko Kachi:** Writing – review & editing. **Akizumi Tsutsumi:** Writing – review & editing, Supervision, Funding acquisition.

## Data statement

The data underlying this article will be shared on reasonable request to the corresponding author.

## Funding

This study was supported by Japan Society for the Promotion of Science (JSPS KAKENHI: Grant Numbers JP20K10477, JP23K09754, and JP24K13538) and Ministry of Health, Labour and Welfare, Japan (Industrial Disease Clinical Research Grants: Grant Numbers 200401-01 and 230201-01).

## Conflicts of interest

The authors declare no conflict of interests.
