# Supplementary material for: Moderating Effect of Psychosocial Safety Climate on the Association of Job Demands and Job Resources With Psychological Distress Among Japanese Employees: A Cross-sectional Study
Source: Saf Health Work. 2025 Feb 3;16(2):213–9. doi: 10.1016/j.shaw.2025.02.001 (PMC12191007; doi:10.1016/j.shaw.2025.02.001)
Supplement: Multimedia component 1 [file mmc1.pdf]

Supplementary Table S1

Associations of demographic and occupational characteristics, job demands, job resources, and psychosocial safety climate (PSC) with nervousness (K6 scale Q1): hierarchical multiple regression analyses

Standardized coefficient (*β*)	Step 1		Step 2		Step 3	
	Estimate	*p*	Estimate	*p*	Estimate	*p*
Age	−0.150	<0.001	−0.158	<0.001	−0.157	<0.001
Gender (men vs. women)	−0.033	0.171	−0.031	0.184	−0.028	0.223
Education (vs. high school or junior high school)						
Graduate school	−0.033	0.163	−0.014	0.517	−0.015	0.496
College	−0.019	0.486	0.013	0.620	0.011	0.678
Junior college	0.019	0.437	0.015	0.521	0.013	0.578
Vocational school	−0.030	0.221	−0.036	0.118	−0.038	0.099
Occupation (vs. managerial employee)						
Non-manual employee	0.029	0.395	−0.022	0.497	−0.023	0.491
Manual employee	0.024	0.445	−0.021	0.469	−0.023	0.428
Other	−0.032	0.227	−0.061	0.016	−0.063	0.013
Work form (vs. day shift)						
Shift work with night duty	0.030	0.164	0.020	0.321	0.020	0.324
Shift work without night duty	0.029	0.176	0.007	0.723	0.008	0.690
Night shift	0.006	0.781	−0.006	0.758	−0.007	0.735
Working hours per week (vs. 30 hours or less)						
31–40 hours	−0.009	0.746	−0.020	0.446	−0.019	0.462
41–50 hours	0.031	0.287	−0.015	0.574	−0.015	0.590
51–60 hours	0.048	0.054	0.007	0.764	0.007	0.774
61 hours or more	0.054	0.021	0.002	0.927	0.001	0.956
Psychological demands			0.193	<0.001	0.187	<0.001
Job control			−0.010	0.669	−0.013	0.582
Supervisor support			−0.049	0.099	−0.050	0.105
Coworker support			−0.022	0.413	−0.011	0.696
Extrinsic reward			−0.206	<0.001	−0.202	<0.001
Psychosocial safety climate (PSC)			−0.021	0.428	−0.021	0.435
Psychological demands × PSC					−0.039	0.065
Job control × PSC					−0.016	0.472
Supervisor support × PSC					−0.016	0.624
Coworker support × PSC					0.038	0.227
Extrinsic reward × PSC					0.008	0.742
Model fit indices	Estimate	*p*	Estimate	*p*	Estimate	*p*
R^2	0.039	—	0.159	—	0.161	—
Adjusted R^2	0.032	—	0.150	—	0.151	—
ΔR^2	0.039	<0.001	0.120	<0.001	0.002	0.280
Residual analysis (Durbin–Watson statistic)	2.053		2.027		2.030	

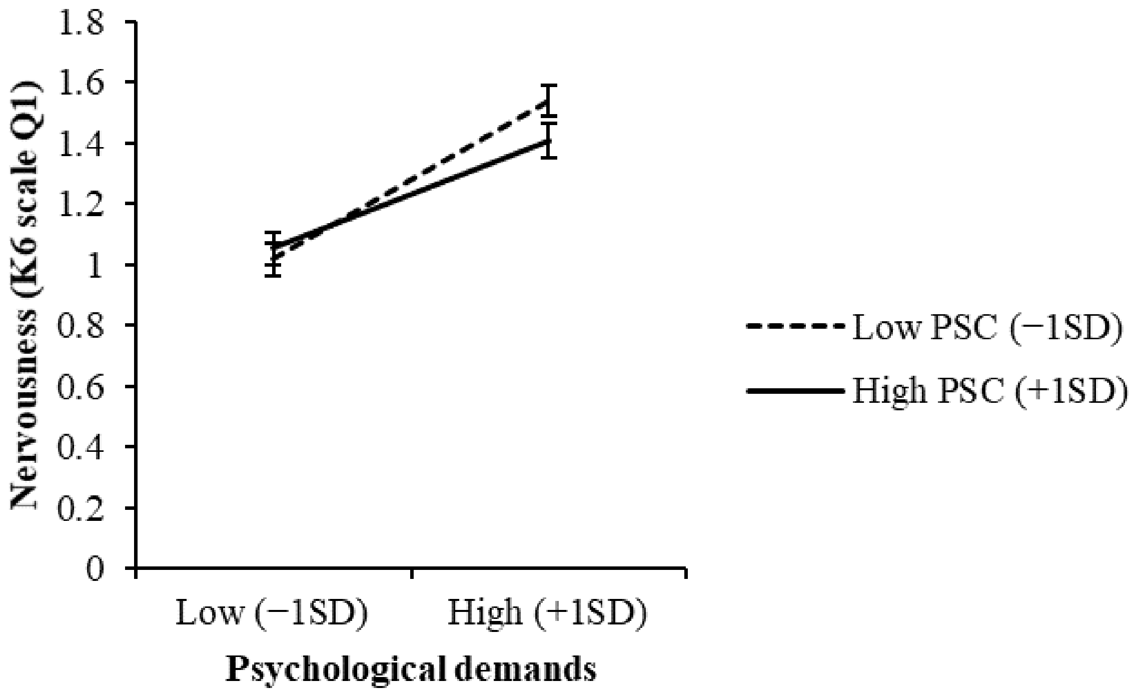

Supplementary Fig. S1. Interaction between psychological demands and psychosocial safety climate (PSC) on nervousness (K6 scale Q1): *post hoc* simple slope analysis. SD, standard deviation.

Supplementary Table S2

Associations of demographic and occupational characteristics, job demands, job resources, and psychosocial safety climate (PSC) with hopelessness (K6 scale Q2): hierarchical multiple regression analyses

Standardized coefficient (*β*)	Step 1		Step 2		Step 3	
	Estimate	*p*	Estimate	*p*	Estimate	*p*
Age	−0.204	<0.001	−0.232	<0.001	−0.230	<0.001
Gender (men vs. women)	0.067	0.006	0.061	0.007	0.064	0.004
Education (vs. high school or junior high school)						
Graduate school	−0.024	0.306	−0.005	0.825	−0.006	0.774
College	−0.031	0.263	0.011	0.653	0.009	0.727
Junior college	0.006	0.811	0.004	0.867	0.001	0.963
Vocational school	−0.015	0.542	−0.020	0.376	−0.023	0.309
Occupation (vs. managerial employee)						
Non-manual employee	0.024	0.480	−0.049	0.129	−0.046	0.149
Manual employee	0.028	0.351	−0.029	0.318	−0.029	0.307
Other	−0.005	0.836	−0.044	0.074	−0.045	0.066
Work form (vs. day shift)						
Shift work with night duty	0.013	0.553	0.011	0.567	0.012	0.564
Shift work without night duty	0.017	0.424	−0.006	0.755	−0.006	0.775
Night shift	0.025	0.224	0.012	0.539	0.011	0.563
Working hours per week (vs. 30 hours or less)						
31–40 hours	−0.004	0.897	−0.025	0.323	−0.024	0.335
41–50 hours	0.024	0.405	−0.020	0.462	−0.020	0.463
51–60 hours	0.059	0.017	0.025	0.283	0.025	0.285
61 hours or more	0.055	0.017	0.008	0.707	0.007	0.745
Psychological demands			0.101	<0.001	0.092	<0.001
Job control			0.011	0.613	0.009	0.676
Supervisor support			−0.014	0.617	−0.019	0.530
Coworker support			−0.034	0.193	−0.016	0.577
Extrinsic reward			−0.275	<0.001	−0.268	<0.001
Psychosocial safety climate (PSC)			−0.082	0.002	−0.084	0.001
Psychological demands × PSC					−0.051	0.014
Job control × PSC					−0.005	0.837
Supervisor support × PSC					−0.033	0.305
Coworker support × PSC					0.058	0.061
Extrinsic reward × PSC					0.020	0.421
Model fit indices	Estimate	*p*	Estimate	*p*	Estimate	*p*
R^2	0.060	—	0.197	—	0.201	—
Adjusted R^2	0.053	—	0.189	—	0.191	—
ΔR^2	0.060	<0.001	0.137	<0.001	0.005	0.027
Residual analysis (Durbin–Watson statistic)	2.026		1.994		2.001	

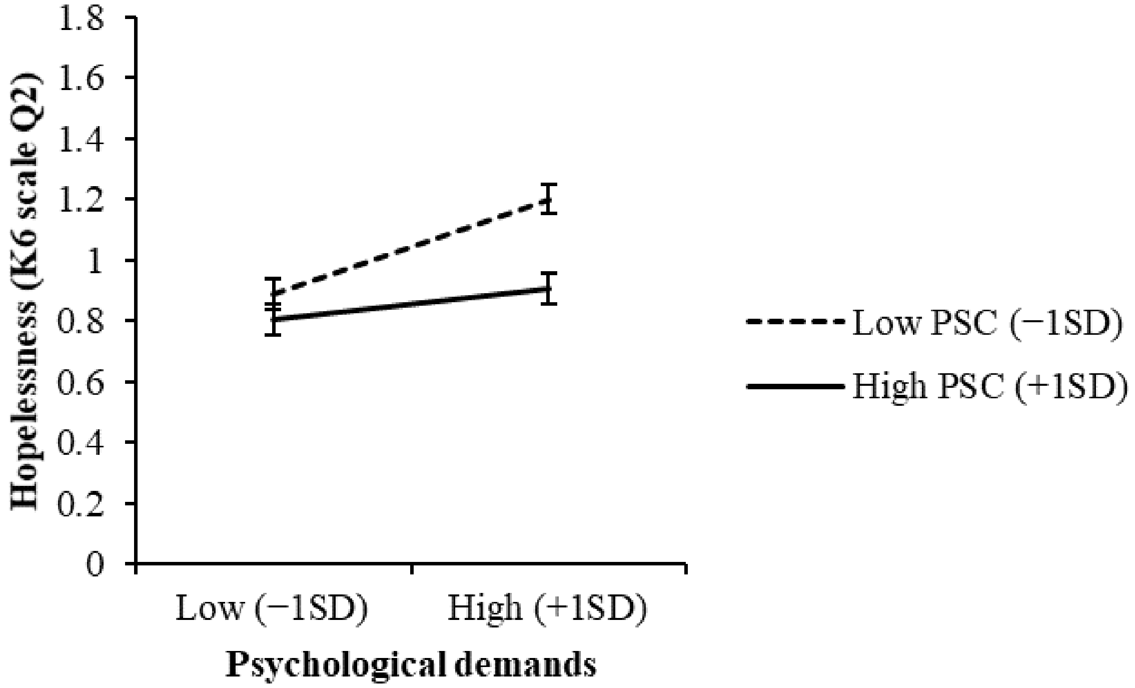

Supplementary Fig. S2. Interaction between psychological demands and psychosocial safety climate (PSC) on hopelessness (K6 scale Q2): *post hoc* simple slope analysis. SD, standard deviation.

Supplementary Table S3

Associations of demographic and occupational characteristics, job demands, job resources, and psychosocial safety climate (PSC) with restlessness/fidgetiness (K6 scale Q3): hierarchical multiple regression analyses

Standardized coefficient (*β*)	Step 1		Step 2		Step 3	
	Estimate	*p*	Estimate	*p*	Estimate	*p*
Age	−0.188	<0.001	−0.195	<0.001	−0.195	<0.001
Gender (men vs. women)	0.030	0.210	0.033	0.147	0.035	0.126
Education (vs. high school or junior high school)						
Graduate school	−0.003	0.881	0.010	0.654	0.010	0.660
College	0.023	0.399	0.052	0.044	0.050	0.054
Junior college	0.031	0.199	0.026	0.252	0.025	0.282
Vocational school	−0.011	0.647	−0.017	0.450	−0.018	0.433
Occupation (vs. managerial employee)						
Non-manual employee	0.033	0.332	−0.015	0.642	−0.017	0.608
Manual employee	0.023	0.443	−0.016	0.582	−0.019	0.513
Other	0.009	0.746	−0.020	0.440	−0.021	0.410
Work form (vs. day shift)						
Shift work with night duty	0.016	0.455	0.010	0.630	0.009	0.646
Shift work without night duty	0.007	0.739	−0.012	0.528	−0.011	0.580
Night shift	0.013	0.524	0.004	0.846	0.003	0.874
Working hours per week (vs. 30 hours or less)						
31–40 hours	0.006	0.823	−0.009	0.738	−0.007	0.773
41–50 hours	0.057	0.046	0.012	0.659	0.014	0.604
51–60 hours	0.098	<0.001	0.059	0.013	0.059	0.013
61 hours or more	0.062	0.007	0.014	0.527	0.014	0.534
Psychological demands			0.160	<0.001	0.153	<0.001
Job control			0.011	0.629	0.009	0.704
Supervisor support			−0.033	0.261	−0.030	0.331
Coworker support			0.013	0.622	0.015	0.604
Extrinsic reward			−0.207	<0.001	−0.208	<0.001
Psychosocial safety climate (PSC)			−0.058	0.028	−0.056	0.035
Psychological demands × PSC					−0.043	0.041
Job control × PSC					−0.015	0.493
Supervisor support × PSC					0.005	0.871
Coworker support × PSC					0.018	0.558
Extrinsic reward × PSC					−0.022	0.371
Model fit indices	Estimate	*p*	Estimate	*p*	Estimate	*p*
R^2	0.061	—	0.161	—	0.164	—
Adjusted R^2	0.054	—	0.153	—	0.153	—
ΔR^2	0.061	<0.001	0.101	<0.001	0.002	0.319
Residual analysis (Durbin–Watson statistic)	2.049		2.053		2.053	

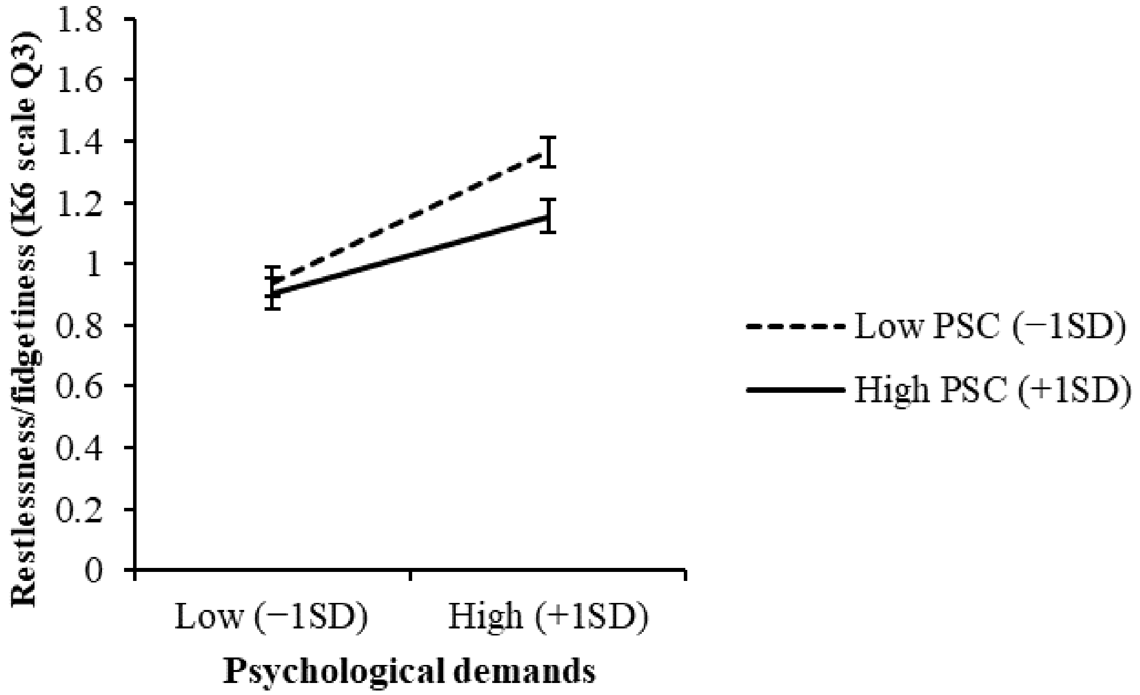

Supplementary Fig. S3. Interaction between psychological demands and psychosocial safety climate (PSC) on restlessness/fidgetiness (K6 scale Q3): *post hoc* simple slope analysis. SD, standard deviation.

Supplementary Table S4

Associations of demographic and occupational characteristics, job demands, job resources, and psychosocial safety climate (PSC) with depression (K6 scale Q4): hierarchical multiple regression analyses

Standardized coefficient (*β*)	Step 1		Step 2		Step 3	
	Estimate	*p*	Estimate	*p*	Estimate	*p*
Age	−0.213	<0.001	−0.230	<0.001	−0.230	<0.001
Gender (men vs. women)	0.002	0.933	0.002	0.922	0.005	0.824
Education (vs. high school or junior high school)						
Graduate school	−0.021	0.364	0.000	0.993	0.000	0.988
College	−0.022	0.414	0.019	0.445	0.016	0.525
Junior college	0.009	0.714	0.006	0.792	0.004	0.869
Vocational school	−0.046	0.056	−0.051	0.023	−0.052	0.019
Occupation (vs. managerial employee)						
Non-manual employee	0.008	0.813	−0.063	0.048	−0.064	0.045
Manual employee	0.013	0.668	−0.046	0.104	−0.049	0.088
Other	−0.011	0.668	−0.050	0.042	−0.052	0.035
Work form (vs. day shift)						
Shift work with night duty	0.027	0.202	0.023	0.250	0.023	0.243
Shift work without night duty	0.017	0.410	−0.007	0.708	−0.006	0.765
Night shift	0.014	0.493	0.001	0.943	0.001	0.979
Working hours per week (vs. 30 hours or less)						
31–40 hours	0.028	0.298	0.011	0.657	0.012	0.625
41–50 hours	0.074	0.009	0.031	0.239	0.032	0.224
51–60 hours	0.093	<0.001	0.057	0.013	0.057	0.013
61 hours or more	0.060	0.009	0.012	0.568	0.012	0.585
Psychological demands			0.139	<0.001	0.130	<0.001
Job control			−0.017	0.453	−0.019	0.389
Supervisor support			−0.014	0.618	−0.014	0.633
Coworker support			−0.007	0.776	−0.005	0.852
Extrinsic reward			−0.268	<0.001	−0.264	<0.001
Psychosocial safety climate (PSC)			−0.078	0.002	−0.075	0.004
Psychological demands × PSC					−0.055	0.008
Job control × PSC					−0.015	0.497
Supervisor support × PSC					−0.017	0.606
Coworker support × PSC					0.018	0.556
Extrinsic reward × PSC					0.009	0.699
Model fit indices	Estimate	*p*	Estimate	*p*	Estimate	*p*
R^2	0.067	—	0.207	—	0.210	—
Adjusted R^2	0.060	—	0.199	—	0.200	—
ΔR^2	0.067	<0.001	0.140	<0.001	0.003	0.119
Residual analysis (Durbin–Watson statistic)	1.985		1.972		1.976	

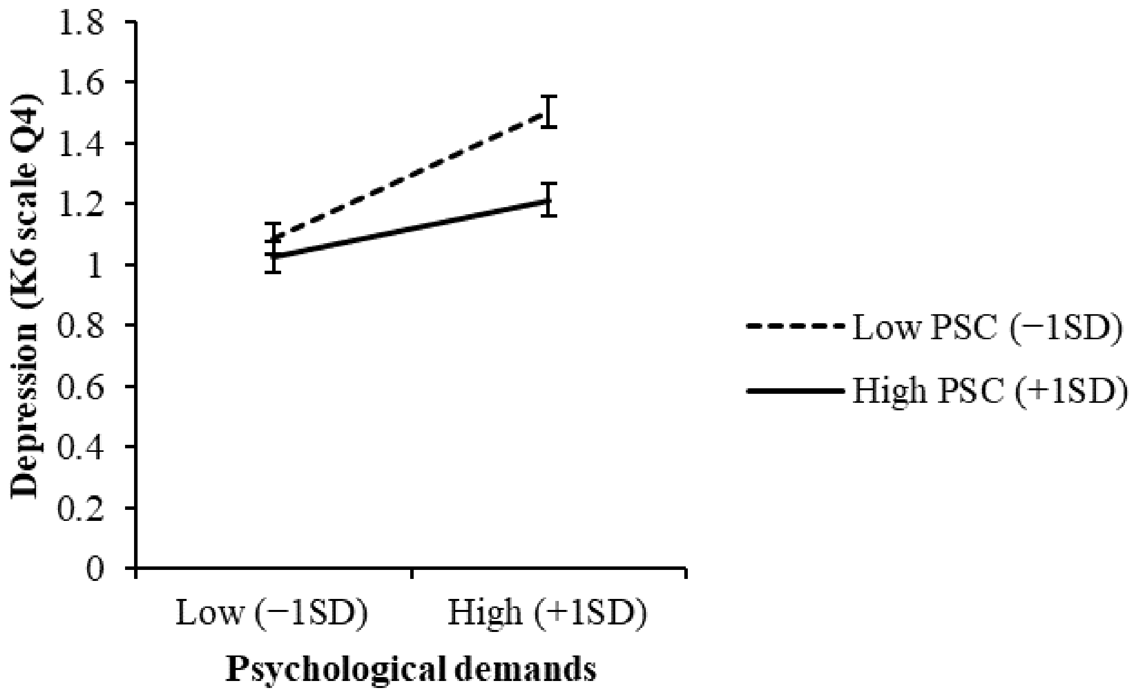

Supplementary Fig. S4. Interaction between psychological demands and psychosocial safety climate (PSC) on depression (K6 scale Q4): *post hoc* simple slope analysis. SD, standard deviation.

Supplementary Table S5

Associations of demographic and occupational characteristics, job demands, job resources, and psychosocial safety climate (PSC) with effortfulness (K6 scale Q5): hierarchical multiple regression analyses

Standardized coefficient (*β*)	Step 1		Step 2		Step 3	
	Estimate	*p*	Estimate	*p*	Estimate	*p*
Age	−0.165	<0.001	−0.182	<0.001	−0.182	<0.001
Gender (men vs. women)	0.010	0.678	0.011	0.633	0.013	0.549
Education (vs. high school or junior high school)						
Graduate school	0.001	0.963	0.021	0.333	0.020	0.346
College	−0.015	0.575	0.026	0.302	0.023	0.357
Junior college	0.002	0.938	−0.001	0.954	−0.003	0.881
Vocational school	−0.026	0.284	−0.033	0.141	−0.034	0.122
Occupation (vs. managerial employee)						
Non-manual employee	0.015	0.664	−0.056	0.079	−0.057	0.074
Manual employee	0.031	0.308	−0.027	0.346	−0.029	0.311
Other	0.012	0.655	−0.028	0.255	−0.030	0.224
Work form (vs. day shift)						
Shift work with night duty	0.064	0.003	0.058	0.004	0.058	0.003
Shift work without night duty	0.018	0.378	−0.007	0.732	−0.005	0.781
Night shift	0.005	0.815	−0.009	0.653	−0.009	0.623
Working hours per week (vs. 30 hours or less)						
31–40 hours	0.019	0.486	0.000	0.993	0.001	0.969
41–50 hours	0.050	0.080	−0.001	0.974	−0.001	0.984
51–60 hours	0.075	0.003	0.031	0.175	0.031	0.179
61 hours or more	0.055	0.018	−0.001	0.980	−0.001	0.958
Psychological demands			0.166	<0.001	0.157	<0.001
Job control			0.004	0.844	0.001	0.956
Supervisor support			−0.017	0.557	−0.016	0.592
Coworker support			−0.024	0.348	−0.024	0.394
Extrinsic reward			−0.260	<0.001	−0.255	<0.001
Psychosocial safety climate (PSC)			−0.096	<0.001	−0.093	<0.001
Psychological demands × PSC					−0.047	0.021
Job control × PSC					−0.017	0.435
Supervisor support × PSC					−0.015	0.641
Coworker support × PSC					0.011	0.718
Extrinsic reward × PSC					0.021	0.381
Model fit indices	Estimate	*p*	Estimate	*p*	Estimate	*p*
R^2	0.048	—	0.206	—	0.209	—
Adjusted R^2	0.041	—	0.198	—	0.199	—
ΔR^2	0.048	<0.001	0.158	<0.001	0.003	0.165
Residual analysis (Durbin–Watson statistic)	2.017		1.985		1.985	

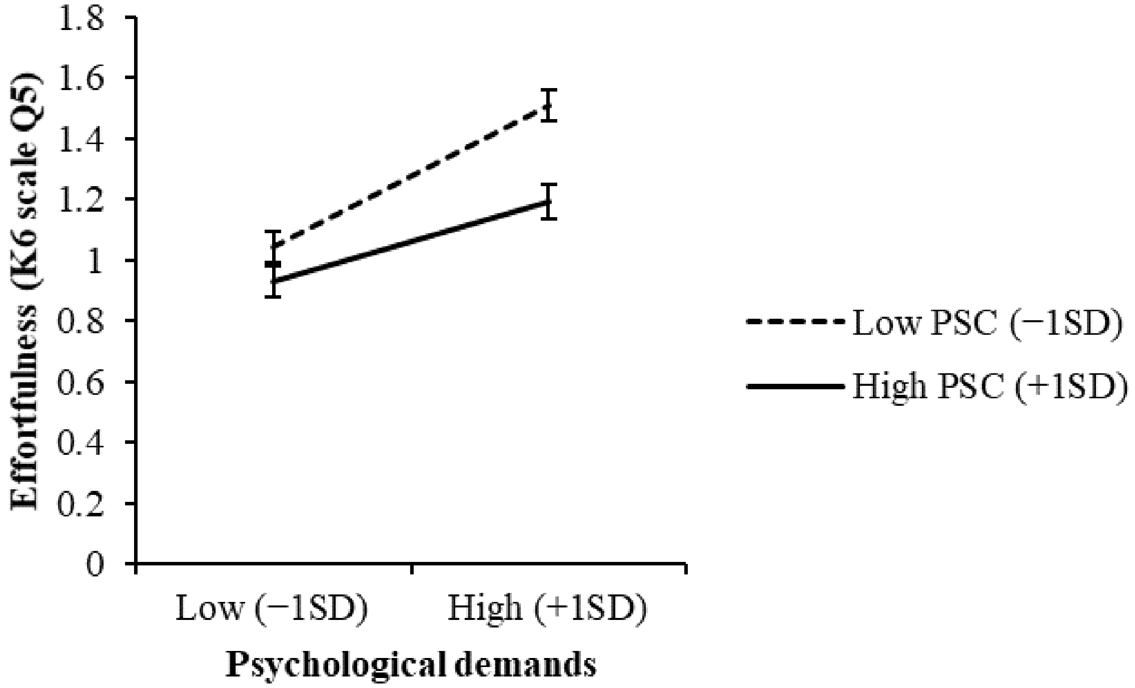

Supplementary Fig. S5. Interaction between psychological demands and psychosocial safety climate (PSC) on effortfulnes (K6 scale Q5): *post hoc* simple slope analysis. SD, standard deviation.

Supplementary Table S6

Associations of demographic and occupational characteristics, job demands, job resources, and psychosocial safety climate (PSC) with worthless (K6 scale Q6): hierarchical multiple regression analyses

Standardized coefficient (β)	Step 1		Step 2		Step 3	
	Estimate	p	Estimate	p	Estimate	p
Age	−0.245	<0.001	−0.268	<0.001	−0.267	<0.001
Gender (men vs. women)	0.054	0.024	0.050	0.029	0.051	0.025
Education (vs. high school or junior high school)						
Graduate school	−0.032	0.167	−0.013	0.549	−0.014	0.538
College	−0.045	0.100	−0.007	0.796	−0.008	0.762
Junior college	−0.012	0.606	−0.013	0.557	−0.015	0.511
Vocational school	−0.009	0.700	−0.012	0.586	−0.013	0.566
Occupation (vs. managerial employee)						
Non-manual employee	0.027	0.424	−0.041	0.200	−0.041	0.211
Manual employee	0.037	0.220	−0.019	0.504	−0.021	0.482
Other	0.012	0.636	−0.023	0.365	−0.023	0.353
Work form (vs. day shift)						
Shift work with night duty	−0.003	0.899	−0.003	0.873	−0.004	0.856
Shift work without night duty	0.008	0.709	−0.012	0.527	−0.012	0.557
Night shift	0.011	0.594	0.000	0.990	0.000	0.990
Working hours per week (vs. 30 hours or less)						
31–40 hours	−0.004	0.890	−0.021	0.403	−0.020	0.434
41–50 hours	0.015	0.591	−0.016	0.564	−0.014	0.607
51–60 hours	0.067	0.006	0.043	0.066	0.043	0.065
61 hours or more	0.014	0.551	−0.021	0.350	−0.021	0.345
Psychological demands			0.075	<0.001	0.069	0.002
Job control			−0.021	0.359	−0.020	0.381
Supervisor support			0.038	0.197	0.032	0.297
Coworker support			−0.033	0.207	−0.023	0.420
Extrinsic reward			−0.265	<0.001	−0.266	<0.001
Psychosocial safety climate (PSC)			−0.071	0.007	−0.072	0.006
Psychological demands × PSC					−0.044	0.037
Job control × PSC					0.004	0.860
Supervisor support × PSC					−0.026	0.425
Coworker support × PSC					0.037	0.237
Extrinsic reward × PSC					−0.019	0.446
Model fit indices	Estimate	p	Estimate	p	Estimate	p
R^2	0.073	—	0.176	—	0.178	—
Adjusted R^2	0.066	—	0.168	—	0.168	—
ΔR^2	0.073	<0.001	0.103	<0.001	0.002	0.357
Residual analysis (Durbin–Watson statistic)	2.043		2.016		2.016	

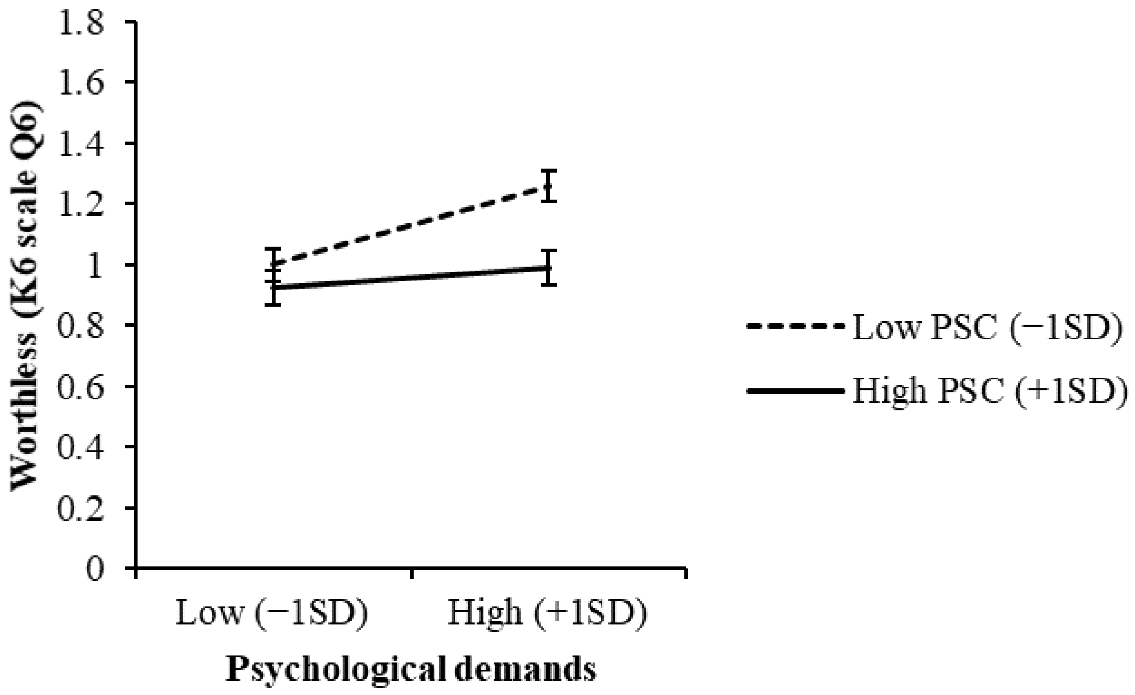

Supplementary Fig. S6. Interaction between psychological demands and psychosocial safety climate (PSC) on worthless (K6 scale Q6): *post hoc* simple slope analysis. SD, standard deviation.
